# Out-of-pocket expenditure on medicine for chronic patients and its economic impact on Indian households: a systematic review and meta-analysis

**DOI:** 10.3389/fmed.2026.1818895

**Published:** 2026-09-04

**Authors:** Deepak Kumar Vaishnav, A. Venkat Raman, Akriti Bansal, Pradeep Nair, Krishna Kharwar

**Affiliations:** 1 Department of New Media, School of Journalism, Mass Communication and New Media, Central University of Himachal Pradesh, Dharamshala, India; 2 Faculty of Management Studies, University of Delhi, North Campus, New Delhi, India

**Keywords:** chronic patients, economic burden, household income, India, medicine, out-of-pocket expenditure

## Abstract

**Background:**

Out-of-pocket expenditure (OOPE) on medicines constitutes a substantial component of healthcare spending in India, particularly among patients with chronic diseases requiring continuous treatment. This systematic review and meta-analysis aimed to synthesise evidence on medicine-related OOPE in India and assess its magnitude and variation across disease categories, healthcare settings, and provider sectors.

**Methods:**

We conducted a systematic review and meta-analysis in accordance with PRISMA 2020 guidelines, with the protocol registered in PROSPERO (CRD420251018386). PubMed, Scopus, and Web of Science were searched for peer-reviewed studies published from 1 January 2008 to 31 March 2025. Two reviewers independently screened studies, extracted data, and assessed methodological quality using Joanna Briggs Institute Critical Appraisal tools. Monetary estimates were standardised to constant 2024 Indian Rupees (INR) using the Consumer Price Index. Random-effects meta-analyses were performed for compatible outcomes, while disease-specific estimates derived from the same study population were synthesised descriptively to avoid treating correlated observations as independent.

**Findings:**

Thirty studies were included in the systematic review, of which 11 contributed to the quantitative meta-analysis. Among patients with chronic diseases, the pooled mean annual medicine-related OOPE was ₹9,550.58 per patient (95% CI: ₹3,642.60–₹15,458.57), with substantial between-study heterogeneity (*I*^2^ = 99.2%). The pooled mean was numerically higher for chronic than non-chronic conditions (₹9,550.58 vs. ₹4,383.79), although the subgroup difference was not statistically significant (*p* = 0.0989). Approximately 51% of individuals incurred medicine-related OOPE (95% CI: 43–59%). Among inpatients, pooled medicine OOPE per episode was ₹3,271.20 (95% CI: ₹852.31–₹5,690.10) in public facilities and ₹4,135.81 (95% CI: −₹2,244.42–₹10,516.04) in private facilities, with no statistically significant subgroup difference (*p* = 0.8039). Medicines accounted for approximately 30–50% of total OOPE during inpatient care and 60–80% during outpatient care. In the disease-specific analysis, four independent estimates of medicine-related OOPE among patients with diabetes were quantitatively pooled, yielding a pooled mean of ₹6,510.20 per patient (95% CI: ₹1,994.35–₹11,026.05), with substantial heterogeneity (*I*^2^ = 99.3%). Estimates for rheumatic disease, hypertension, cancer, acid peptic disease, and thyroid disorders were presented descriptively because of limited evidence and non-independent estimates from the same study population.

**Interpretation:**

Medicine-related OOPE represents a substantial financial burden in India, particularly for patients requiring long-term treatment for chronic diseases. However, considerable heterogeneity and limited evidence within several subgroup analyses warrant cautious interpretation of pooled estimates. Strengthening public-sector medicine supply, improving access to affordable essential and generic medicines, and extending financial protection to outpatient medicines may help reduce household financial burden and advance Universal Health Coverage in India.

**Systematic review registration:**

https://www.crd.york.ac.uk/PROSPERO/view/CRD420251018386, PROSPERO registration: CRD420251018386.

## Introduction

OOPE on healthcare creates a significant burden on households especially in low- and middle-income countries, including India, where it accounts for a substantial proportion of overall health financing (1). OOPE on healthcare often increases significantly, compelling families to divert funds from basic needs such as food, education, and housing resulting in Catastrophic health expenditure (CHE) (2).

Globally, non-communicable diseases are the major cause of death, a large part of which occurs in low- and middle-income countries (3). The risk of death in elderly people is significantly increased because the prevalence of these conditions rises dramatically with age (4). In India, multi-morbidity is strongly linked to ageing, and older people, in particular those from disadvantaged socio-economic groups, are especially hard hit by the burden of these conditions, which often results in avoidance of treatment because of unaffordability (5, 35, 40).

The increased chronic illnesses, a weak financial safety net, and excessive dependence on OOP payments are significant problems in the Indian healthcare system (6, 28). Among patients with chronic conditions, medicines take the biggest portion of OOPE on healthcare (7). The increase in government initiatives and the focus on providing basic medicines at zero cost in government facilities would significantly alleviate the burden on the poor and protect them from falling further into poverty (8). Despite the introduction of multiple schemes by central and state governments, such as the Pradhan Mantri Bharatiya Janaushadhi Pariyojana (The Prime Minister Indian Generic Medicine Scheme) and other subsidy programmes, OOPE on healthcare remains persistently high amongst households. Poorer households face greater hardship due to OOPE on healthcare as compared to wealthier counterparts. (9).

Within the broader framework of Universal Health Coverage (UHC), financial protection is considered a core objective of health systems, particularly in low- and middle-income countries (LMICs), where households rely heavily on out-of-pocket expenditure (OOPE) to access healthcare services (10).

In India, medicines constitute one of the largest components of OOPE, especially among the patients with chronic diseases who require continuous and long-term treatment. Because outpatient medicines are often inadequately covered under public financing and insurance mechanisms, households frequently bear substantial direct costs for essential medicines (11). High medicine-related OOPE contributes not only to catastrophic health expenditure and medical impoverishment but also to healthcare financing inequality, treatment non-adherence, and reduced access to healthcare among socioeconomically disadvantaged populations. Medicine affordability is therefore increasingly recognised as a central issue in financial risk protection and equitable healthcare financing.

Although previous studies in India have examined overall healthcare OOPE and catastrophic health expenditure, evidence specifically related to medicine-related OOPE remains fragmented across diseases, healthcare settings, and population groups (36). Most studies have focused on aggregate healthcare expenditure without systematically examining how medicine costs contribute to financial vulnerability among chronic disease patients. Furthermore, limited evidence exists regarding variations in medicine-related OOPE across inpatient and outpatient care settings, public and private healthcare sectors, and different chronic disease categories.

By synthesising the available evidence, this systematic review and meta-analysis contributes to the broader literature on healthcare financing inequality, medicine affordability, and financial protection in low- and middle-income countries by providing pooled estimates of medicine-related OOPE in India based on the available evidence, together with contextual analyses across disease categories, healthcare settings, and provider sectors.

The study was undertaken to investigate the following research questions:

1. What is the magnitude of household OOPE spending on medicines among chronically ill patients in India?
2. How is OOPE allocated between chronic and non-chronic disease conditions?
3. How does the burden of household OOPE on medicines vary by healthcare facility type and care setting in India?
4. How does household out-of-pocket spending on medicines vary across different chronic diseases in India?

## Method

### Conceptual framework

This study is conceptually grounded in the broader literature on Universal Health Coverage (UHC), financial protection, healthcare financing inequality, and medicine affordability in low- and middle-income countries (LMICs). In India, chronic diseases require continuous and long-term pharmacological treatment, resulting in sustained medicine expenditure among households. Because outpatient medicines are inadequately covered under many public financing and insurance mechanisms, households frequently rely on out-of-pocket expenditure (OOPE) to access essential medicines.

High medicine-related OOPE contributes to catastrophic health expenditure (CHE), financial hardship, treatment non-adherence, and healthcare inequality, particularly among socioeconomically disadvantaged populations. The burden is further influenced by differences in healthcare settings, disease categories, and public-private healthcare sectors. This review therefore, conceptualises medicine-related OOPE not merely as a measure of healthcare spending, but also as an indicator of gaps in financial protection and equitable healthcare financing in India.

**Figure 1:**
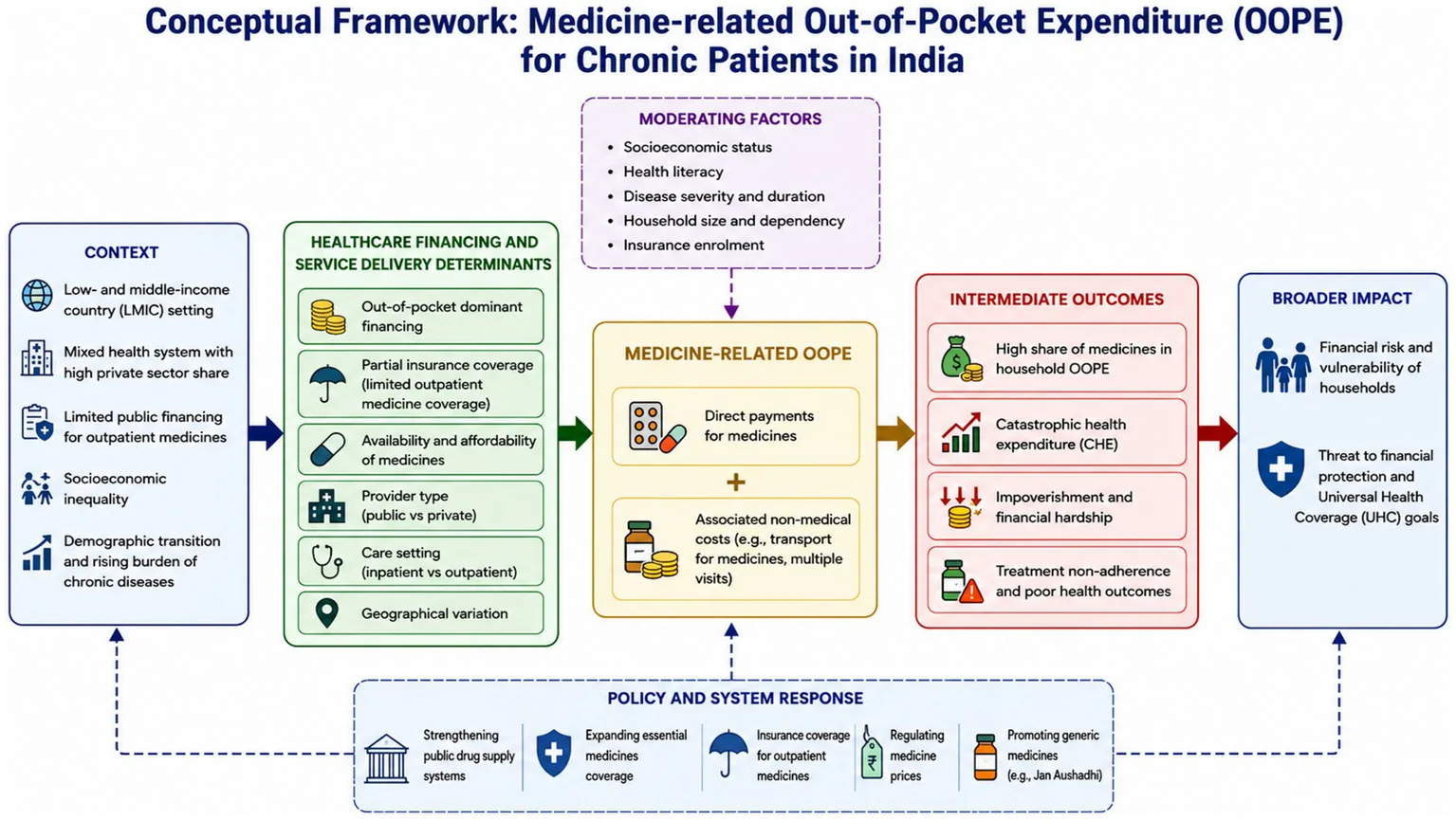
Conceptual framework illustrating the pathways linking chronic diseases, medicine-related out-of-pocket expenditure, healthcare financing, and financial protection in India.

The conceptual framework underpinning this systematic review is presented in Figure 1. It illustrates the conceptual pathways linking chronic diseases, medicine-related out-of-pocket expenditure (OOPE), healthcare financing and service delivery determinants, financial hardship, and the broader implications for financial protection and Universal Health Coverage (UHC). This framework guided the development of the review questions, data extraction, and interpretation of the findings.

We conducted a systematic review and meta-analysis to understand the OOPE on medicines in India for chronic diseases. The review was conducted in accordance with the Preferred Reporting Items for Systematic Reviews and Meta-Analyses (PRISMA) 2020 guidelines. Ethical approval from an Institutional Review Board was not required, as the study was based solely on secondary data and did not involve individual patient information. The review protocol was registered with PROSPERO (CRD420251018386).

### Search strategy and selection criteria

We conducted a systematic search of databases, namely Scopus, PubMed, and Web of Science, to identify peer-reviewed studies reporting OOPE on medicines among patients with chronic conditions in India. The search included articles published between 1 January 2008 to 31 March 2025.

Although the search strategy was primarily designed to identify studies of medicine-related OOPE among patients with chronic conditions, the search also retrieved studies reporting relevant medicine-related OOPE data for non-chronic conditions. Where such data met the predefined eligibility criteria and were extractable, they were retained to enable the planned comparison of medicine-related OOPE between chronic and non-chronic conditions.

The search strategy combined keywords and Medical Subject Headings (MeSH) related to out-of-pocket expenditure, medicines, chronic diseases, healthcare costs, and India using Boolean operators (AND/OR). Searches were adapted for each database, and the detailed search strategy for each is provided in Supplementary File S1.

Search strings used in the database search were:

*(“out-of-pocket” OR “catastrophic health expenditure” OR “OOPE”) AND (“medicine” OR “drug expenditure” OR “*

*pharmaceutical”) AND (“chronic disease” OR “non-communicable disease” OR “diabetes” OR “cardiovascular disease”) AND (“India”)*.

Equivalent search terms and syntax were adapted for PubMed and Web of Science.

### Study eligibility

We have included original studies that (1) were conducted in India; (2) reported quantitative estimates of OOPE on medicines at the household or individual level; (3) involved populations with chronic non-communicable diseases, acute (non-chronic) diseases, or both; and (4) provided sufficient statistical information (such as means, standard deviations, medians, or proportions) to allow for pooled estimation. We excluded studies that did not report identifiable medicine-specific OOPE or reported only total healthcare expenditure or total OOPE without separately reporting expenditure on medicines. Studies involving acute or non-chronic conditions were included when they reported identifiable medicine-specific OOPE estimates, as these data were required for the planned comparison between chronic and non-chronic conditions.

### Data charting and extraction

All records retrieved from the databases were downloaded and de-duplicated by one author using Microsoft Excel, employing conditional formatting and duplicate-value functions. Two authors independently screened and evaluated the titles and abstracts within spreadsheets. Full-text articles deemed potentially eligible were then examined in detail by at least two authors, using the predefined inclusion criteria.

In the final stage, data were independently extracted from the eligible studies by two authors using a standardized spreadsheet template. The extracted variables were authors, year of publication, year of data collection, geographical region, socio-economic characteristics, population type and sample size, disease category, healthcare setting (inpatient or outpatient; public or private), and indicators of medicine-related OOPE, such as mean expenditure, standard deviation, proportions, or the proportion of medicines in total healthcare expenditure.

Disagreements between reviewers were resolved through discussion, and any unresolved issues were mediated by the lead reviewer. Where there was uncertainty regarding data eligibility or extractability, consensus was achieved by consulting the lead reviewer.

Key variables extracted from the systematic review are presented in Supplementary (S5-A), which was derived from the main data extraction sheet. Table 1 summarises study characteristics, population type, disease type, healthcare setting, facility type, and sample size.

**Table 1:** Summary characteristics of studies included in the systematic review (*N* = 30).

Domain	Category	Number of studies	Percentage (%)
Study design	Cross-sectional	27	90.0
	Longitudinal	1	3.3
	Qualitative	1	3.3
	Prospective	1	3.3
Geographic coverage	Nationally representative	13	43.3
	State level	7	23.3
	District level	4	13.3
	City level	2	6.7
	Capital city	1	3.3
	Union Territory	1	3.3
	Regional (multi-state)	1	3.3
	Not reported	1	3.3
Region of India	Northern India	8	26.7
	Eastern India	3	10.0
	Southern India	2	6.7
	Multiple/mixed regions	17	56.7
Area	Rural and urban	23	76.7
	Urban only	5	16.7
	Rural only	1	3.3
	Not reported	1	3.3
Sex distribution	Female only	9	30.0
	Male only	6	20.0
	Both sexes	13	43.3
	Not reported	2	6.7
Age group (years)	18–60 years	25	83.3
	>60 years	4	13.3
	Not reported	1	3.3
Disease category	Diabetes mellitus	8	26.7
	Hypertension	5	16.7
	Diabetes and hypertension	5	16.7
	Cancer	4	13.3
	Chronic diseases (unspecified)	4	13.3
	Mixed acute and chronic conditions	2	6.7
	Acute conditions only	1	3.3
	Joint pain	1	3.3
	Lymphatic filariasis	1	3.3

A map illustrating the geographical distribution of the included studies across India is presented in Supplementary S4. Only studies that reported a specific geographical location within India were included in the map. Nationally representative studies without a specific state-level location were not plotted on the map.

Detailed information on the measurement and reporting of medicine-related OOPE, including the recall period, time reference, currency reported, conversion to Indian rupees, and the type of OOPE measure reported (mean, median, proportion, or mixed), is presented in Supplement (S5-B).

### Definition

OOP payments on medicines are defined as all direct payments made by the patient or household for medicines used in healthcare (12). Chronic diseases are long-term diseases, such as diabetes, cancer, and asthma, that develop slowly and get worse over time (3). In contrast, acute conditions arise abruptly, are accompanied by immediate and fast-developing symptoms, and are usually of limited duration (13). Inpatient care is the medical treatment given when a patient is admitted to the hospital and spends a night or more, while outpatient care is the medical treatment given without requiring the patient to stay in the hospital (14, 45).

### Quality assessment of the study

The methodological quality of the included studies was assessed using the Joanna Briggs Institute (JBI) Critical Appraisal tools according to the study design. Three different JBI Critical Appraisal Checklists were used in the study.

- The JBI Critical Appraisal Checklist for Analytical Cross-Sectional Studies was used to assess the 27 cross-sectional studies.
- The JBI Critical Appraisal Checklist for Cohort Studies was used to assess the longitudinal and prospective cohort studies.
- The JBI Critical Appraisal Checklist for Qualitative Research was used to assess the qualitative study.

Two reviewers independently conducted the quality assessment, and disagreements were resolved through discussion with the lead reviewer.

The key elements of internal and external validity assessed in the study include the clarity of inclusion criteria, the validity and reliability of exposure and outcome measurements, the identification and management of confounding factors, and the appropriateness of statistical analyses. Each study was assigned an overall quality rating based on the extent to which it met the established criteria. Studies that fulfilled less than 50% of the appraisal criteria were considered to be of low methodological quality and were excluded from the quantitative synthesis. A summary of the quality ratings at the study level is provided in the Supplementary materials, along with more detailed item-wise assessments (Supplementary S5-C).

## Statistical analysis

Descriptive analyses were conducted to summarise the methodological characteristics of the included studies, including participant characteristics, disease categories, healthcare settings, facility types, and measures of medicine-related out-of-pocket expenditure (OOPE). Continuous outcomes were summarised using means and standard deviations or medians and interquartile ranges, as reported in the original studies, while categorical variables were summarised using proportions and percentages. Studies reporting extractable numerical estimates of medicine-related OOPE were considered eligible for quantitative synthesis. Although some studies reported medicine-related OOPE as medians with interquartile ranges (IQRs), the number of such studies was limited and reporting formats were heterogeneous. Therefore, a separate meta-analysis of medians was not undertaken. These studies were retained in the systematic review and synthesised narratively where appropriate, while the quantitative meta-analysis was restricted to studies reporting compatible mean-based estimates. Most expenditure estimates were reported in Indian Rupees (INR). Where feasible, expenditure estimates reported over shorter recall periods were annualised to improve comparability. To ensure comparability of cost estimates across studies conducted in different years, all monetary values were standardised to constant 2024 Indian Rupees (INR) before quantitative synthesis. For studies reporting costs in earlier years, both the mean expenditure and standard deviation were adjusted using the Consumer Price Index (CPI) for India. Studies reporting costs in foreign currencies were first converted to Indian Rupees using the exchange rate corresponding to the year of cost estimation and were subsequently adjusted to constant 2024 INR. The detailed inflation adjustment procedure is described in the section “Inflation Adjustment of Monetary Values.

Random-effects meta-analysis models were used because considerable methodological and contextual heterogeneity was anticipated across studies. For continuous outcomes, pooled mean medicine-related OOPE estimates and corresponding 95% confidence intervals (CIs) were calculated using inverse-variance methods under a random-effects model. Mean expenditure estimates were analysed on the original INR scale. Because healthcare expenditure data are typically right-skewed, the pooled mean estimates were interpreted cautiously, particularly in the presence of substantial between-study heterogeneity and extreme expenditure values (31).

Proportions of persons with OOPE related to medicines were estimated in pooled proportions with appropriate variance-stabilising transformations and all pooled estimates are presented with 95% confidence intervals (CIs). Cochran’s Q test was used to evaluate statistical heterogeneity and the *I*^2^ statistic was used to quantify statistical heterogeneity (38).

Where sufficient data were available, pre-specified subgroup analyses were performed to examine potential sources of heterogeneity by disease category (chronic versus non-chronic diseases), healthcare setting (inpatient versus outpatient care), and facility type (public versus private healthcare facilities). Due to differences in outcome units, estimates for inpatient and outpatient services were not pooled directly but were analysed separately. Formal tests for subgroup differences were performed where appropriate, and the results were interpreted cautiously in the presence of substantial heterogeneity. Where a single study reported multiple disease-specific estimates derived from the same study population, these estimates were not treated as independent observations across disease categories. Disease-specific estimates representing the same study population were therefore presented descriptively, except where a prespecified disease-specific estimate could be incorporated into a quantitative synthesis without duplication of participants. Where disease-specific estimates represented distinct, prespecified subgroups with compatible data and were considered analytically independent, they were eligible for disease-specific quantitative synthesis. To assess the robustness of the pooled findings, sensitivity analyses were conducted by excluding studies reporting household-level OOPE estimates, as described in Supplementary S7 (37). Despite the substantial statistical heterogeneity observed across studies, quantitative synthesis was retained because the primary objective of this review was to estimate the overall magnitude and variation of medicine-related OOPE across diverse healthcare settings in India rather than to derive a single common underlying effect. Considerable heterogeneity was anticipated owing to differences in disease profiles, healthcare settings, geographical regions, study populations, recall periods, and costing methods. Therefore, random-effects models were considered appropriate. The pooled estimates should be interpreted as summary measures of the available evidence rather than as precise national estimates and should be considered alongside the descriptive synthesis.

Nationally representative and subnational studies were included together in the meta-analysis because both contributed relevant evidence on medicine-related OOPE in India.

All analyses were conducted using RStudio (version 2025.05.1 + 513), and the pooled data are provided in Supplementary S6 (34).

### Sensitivity analysis

Studies reporting household-level OOPE estimates were excluded from the quantitative pooling because their unit of analysis was not directly comparable with per-patient expenditure estimates; these studies were retained for descriptive synthesis. (Supplementary S7).

### Inflation adjustment of monetary values

**Figure 2:**
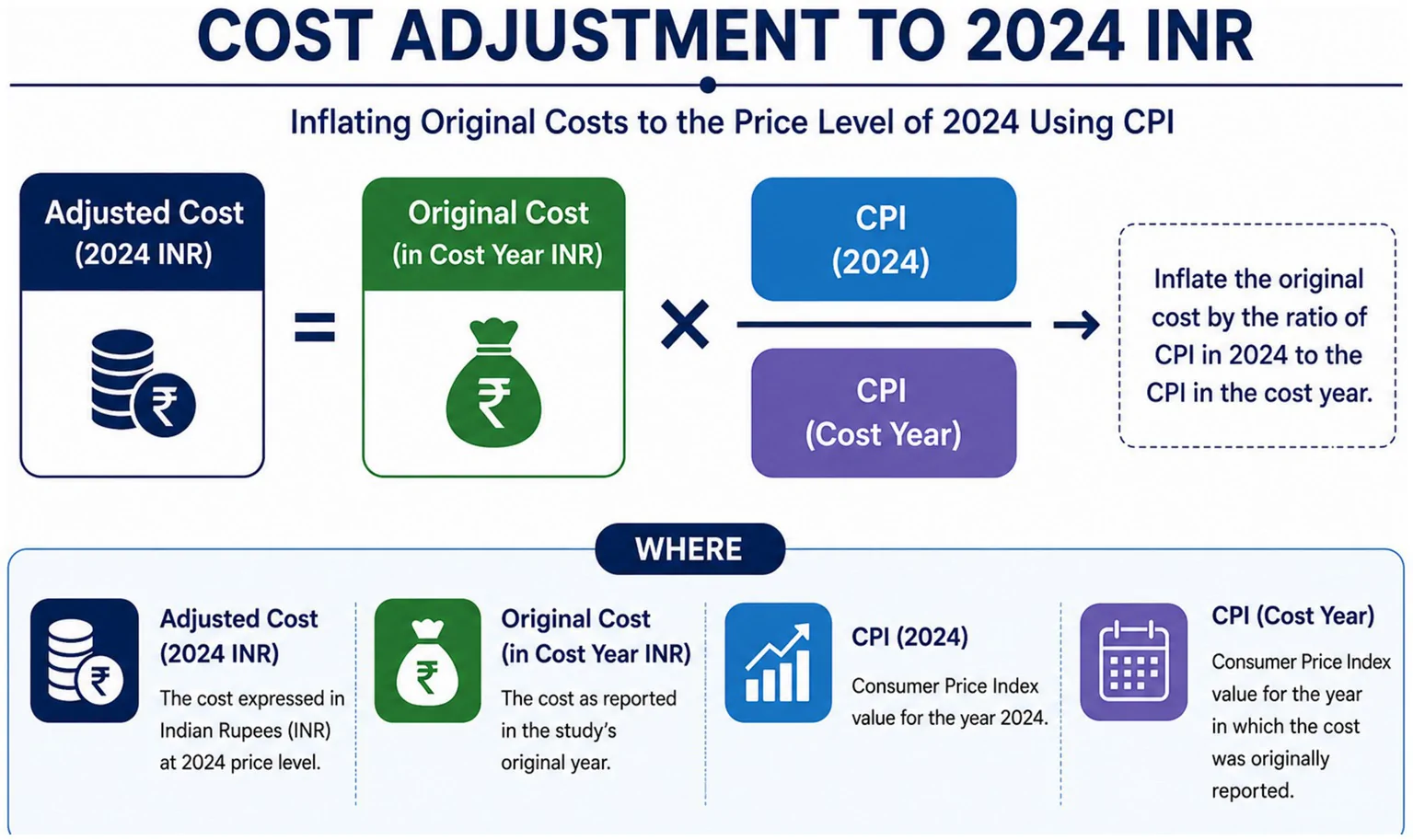
Inflation adjustment of reported costs to 2024 Indian Rupees (INR) using the Consumer Price Index (CPI) (15).

To ensure comparability of cost estimates across studies conducted in different years, all monetary values were standardized to constant 2024 Indian Rupees (INR) using the Consumer Price Index (CPI). For studies reporting medicine-related out-of-pocket expenditure in earlier years, both the reported mean expenditure and standard deviation (SD) were adjusted for inflation using the CPI. Annual average CPI values were obtained from the Consumer Price Index (Combined) published by the Ministry of Statistics and Programme Implementation (MoSPI), Government of India. Where required, the historical CPI series compiled from official MoSPI data was used to derive annual average indices (see Figure 2).

The inflation adjustment was performed using the following formula:

For studies reporting costs in currencies other than Indian Rupees (INR), expenditure values were first converted to INR using the exchange rate reported in the original study. Where an exchange rate was not reported, the average annual exchange rate corresponding to the study cost year was used. The converted INR values were subsequently adjusted for inflation to constant 2024 INR using the corresponding CPI adjustment factor. As all included studies were conducted in India and the objective was to estimate medicine-related OOPE in Indian settings, market exchange rates were used for currency conversion, and purchasing power parity (PPP) adjustments were not applied.

Subsequently, all expenditure estimates (means and standard deviations) were adjusted to constant 2024 Indian Rupees (INR) using the annual average Consumer Price Index (CPI) for India. The inflation adjustment factor was calculated as:

Inflation Factor = CPI2024/CPI Cost year.

Both the reported mean expenditure and standard deviation were multiplied by the corresponding inflation adjustment factor to obtain inflation-adjusted values expressed in constant 2024 INR. The meta-analysis was subsequently repeated using these standardized cost estimates. The year 2024 was selected as the common price year because complete annual CPI data for 2025 were not available at the time the analysis was conducted.

The inflation-adjusted cost estimates were subsequently used in all meta-analyses to improve comparability across studies conducted over different time periods (15, 16).

## Limitation

Substantial heterogeneity across studies was anticipated because of differences in study populations, disease categories, healthcare settings, recall periods, geographical regions, and methods used to estimate medicine-related OOPE. Therefore, random-effects models were selected *a priori*, as they account for both within-study and between-study variability and are considered appropriate for healthcare expenditure meta-analyses involving heterogeneous populations and contexts. The pooled estimates generated in this review should therefore be interpreted as indicative summary patterns rather than precise national estimates.

In addition, healthcare expenditure data are often right-skewed, and the use of mean expenditure estimates on the original scale may have increased the influence of extreme values reported in individual studies. Because mean expenditure estimates were analysed on the original INR scale, the normal-approximation confidence interval for one pooled estimate extended below zero; this should be interpreted as reflecting substantial statistical uncertainty rather than the possibility of negative expenditure.

Another limitation is that pooled analyses combined nationally representative studies with subnational studies conducted in specific states, districts, or healthcare facilities. Regional differences in healthcare access, medicine prices, socioeconomic conditions, and healthcare utilisation may therefore have contributed to variability in pooled estimates.

## Results

### Summary of the study characteristics

**Figure 3:**
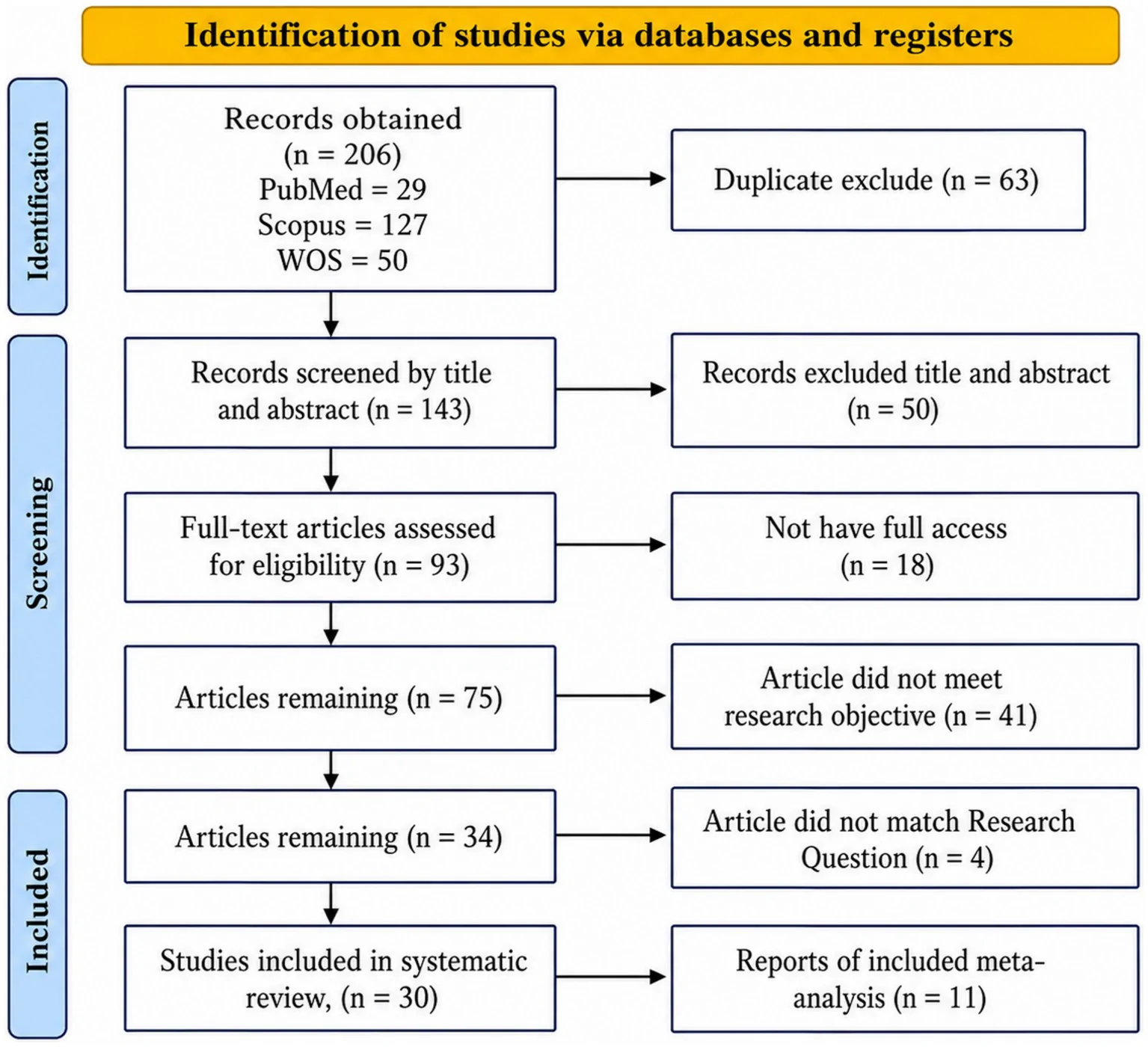
PRISMA flow diagram showing the study selection for the systematic review and meta-analysis (27).

Figure 3 illustrates the identification, screening, and inclusion process of the studies. A total of 206 records were retrieved from Web of Science (*n* = 50), PubMed (*n* = 29), and Scopus (*n* = 127). After removing 63 duplicate records, 143 articles were screened, of which 50 were excluded. The remaining 93 articles were assessed for eligibility. Of these, 18 were excluded because the full text was unavailable. The remaining 75 articles underwent further evaluation, with 41 excluded for not meeting the research objectives. Of the 34 articles that remained, four were excluded as they did not align with the research question. Ultimately, 30 studies were included in the systematic review, of which 11 were incorporated into the meta-analysis.

In this review, 30 included studies, 27 (90.0%) used a cross-sectional design, while one study each (3.3%) used a longitudinal, prospective, and qualitative design. Overall, the evidence base was predominantly cross-sectional, with limited representation of other study designs.

Geographically, 13 studies (43.3%) were nationally representative. Subnational evidence was reported by seven studies (23.3%) at the state level, four studies (13.3%) at the district level, and two studies (6.7%) at the city level. One study each (3.3%) was conducted at the capital-city level, in a Union Territory, and at the regional level. Geographic coverage was not reported in one study (3.3%). Overall, the included studies were more heavily represented by nationally representative data than by subnational or local-level data.

Regarding the regional distribution, eight studies (26.7%) were carried out in Northern India, three studies (10.0%) in Eastern India, and two studies (6.7%) in Southern India. Most of the studies (17; 56.7%) dealt with several geographical regions or mixed geographical areas.

### Subgroup analysis

**Figure 4:**
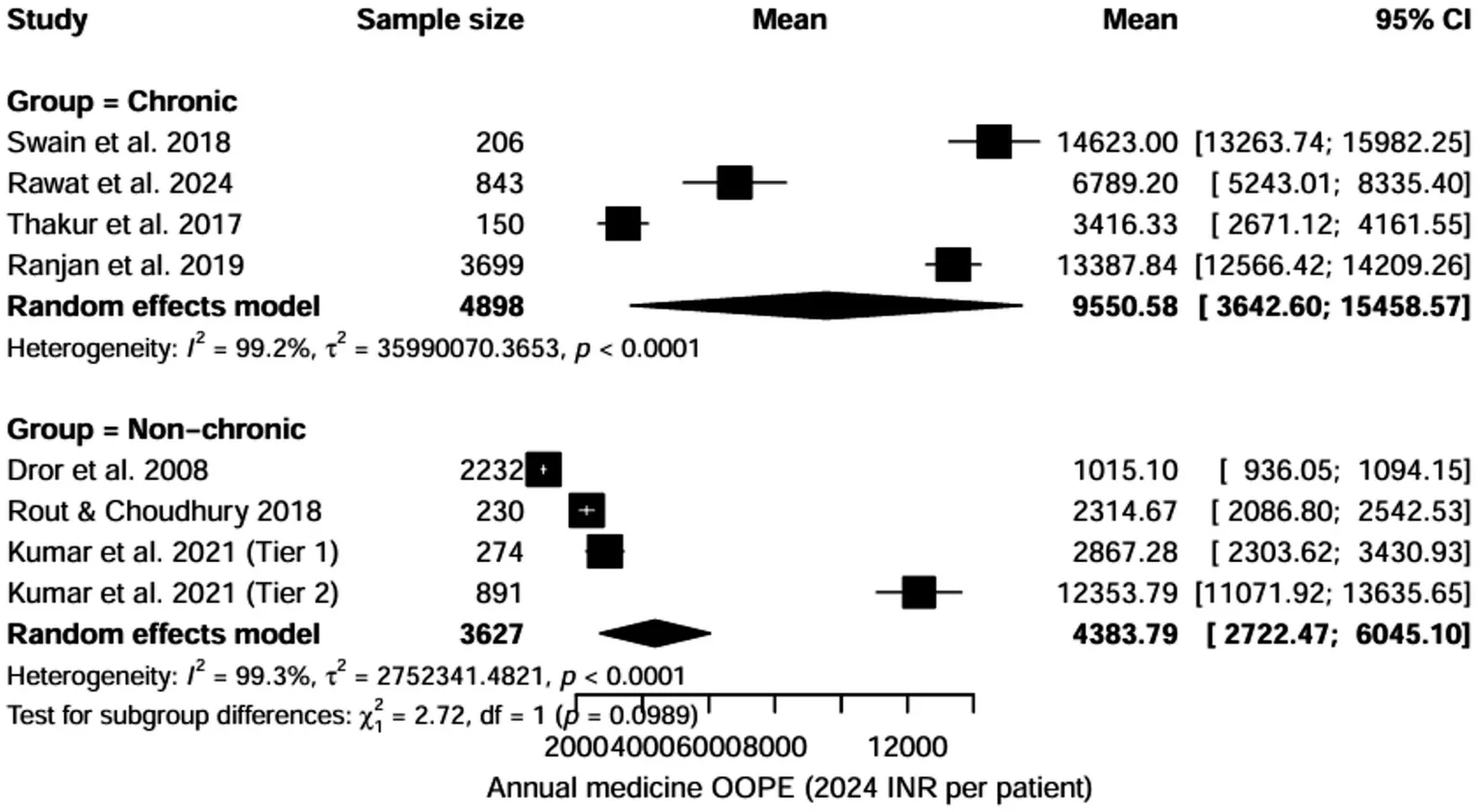
Random-effects subgroup meta-analysis comparing the pooled mean annual out-of-pocket expenditure (OOPE) on medicines among patients with chronic and non-chronic conditions in India based on the available evidence. All costs were inflation-adjusted to 2024 Indian Rupees (INR) using the Consumer Price Index (CPI). Squares represent study-specific mean estimates with 95% confidence intervals (CIs), and diamonds represent the pooled subgroup estimates from the random-effects model.

**Figure 5:**
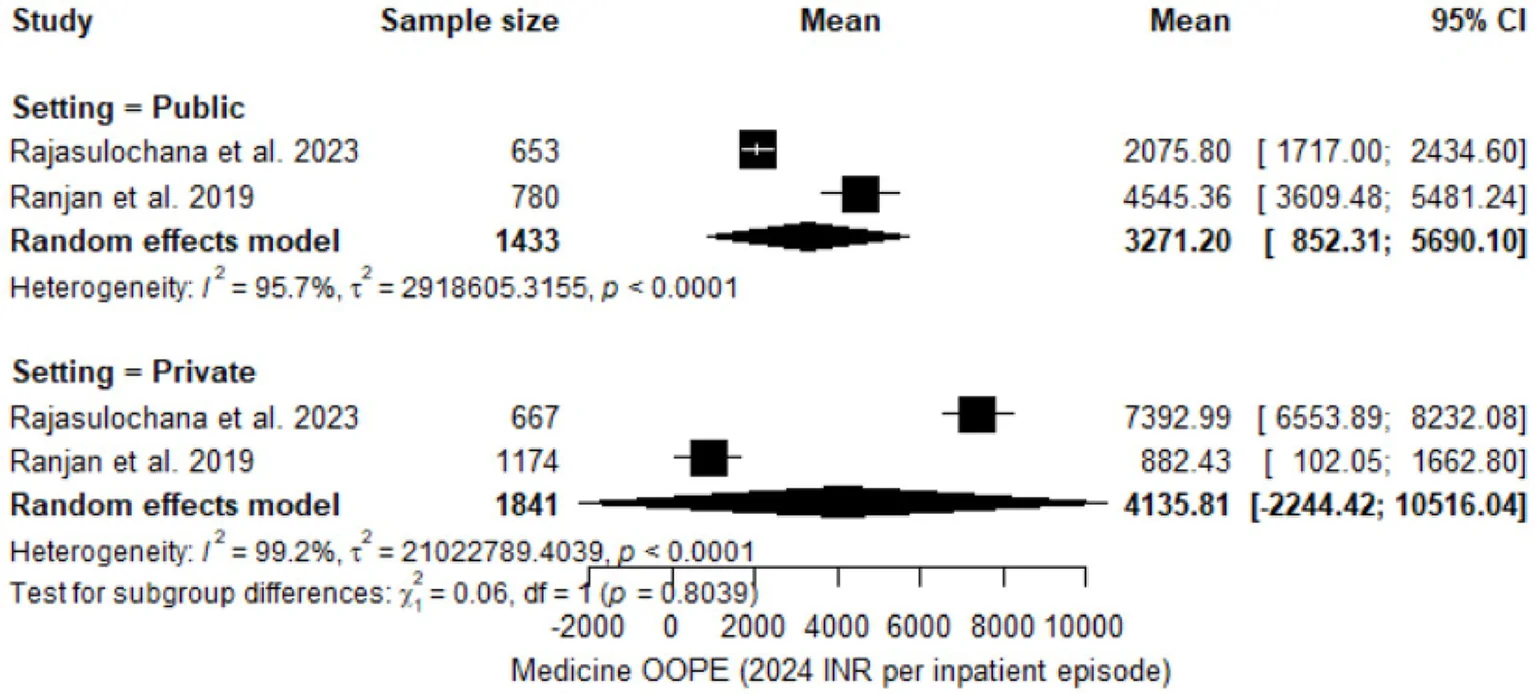
Random-effects forest plot comparing pooled mean medicine out-of-pocket expenditure (OOPE) per inpatient episode between public and private healthcare settings in India. Costs were adjusted to 2024 INR using the Consumer Price Index (CPI). Squares indicate study-specific estimates with 95% confidence intervals, and diamonds indicate pooled subgroup estimates.

**Figure 6:**
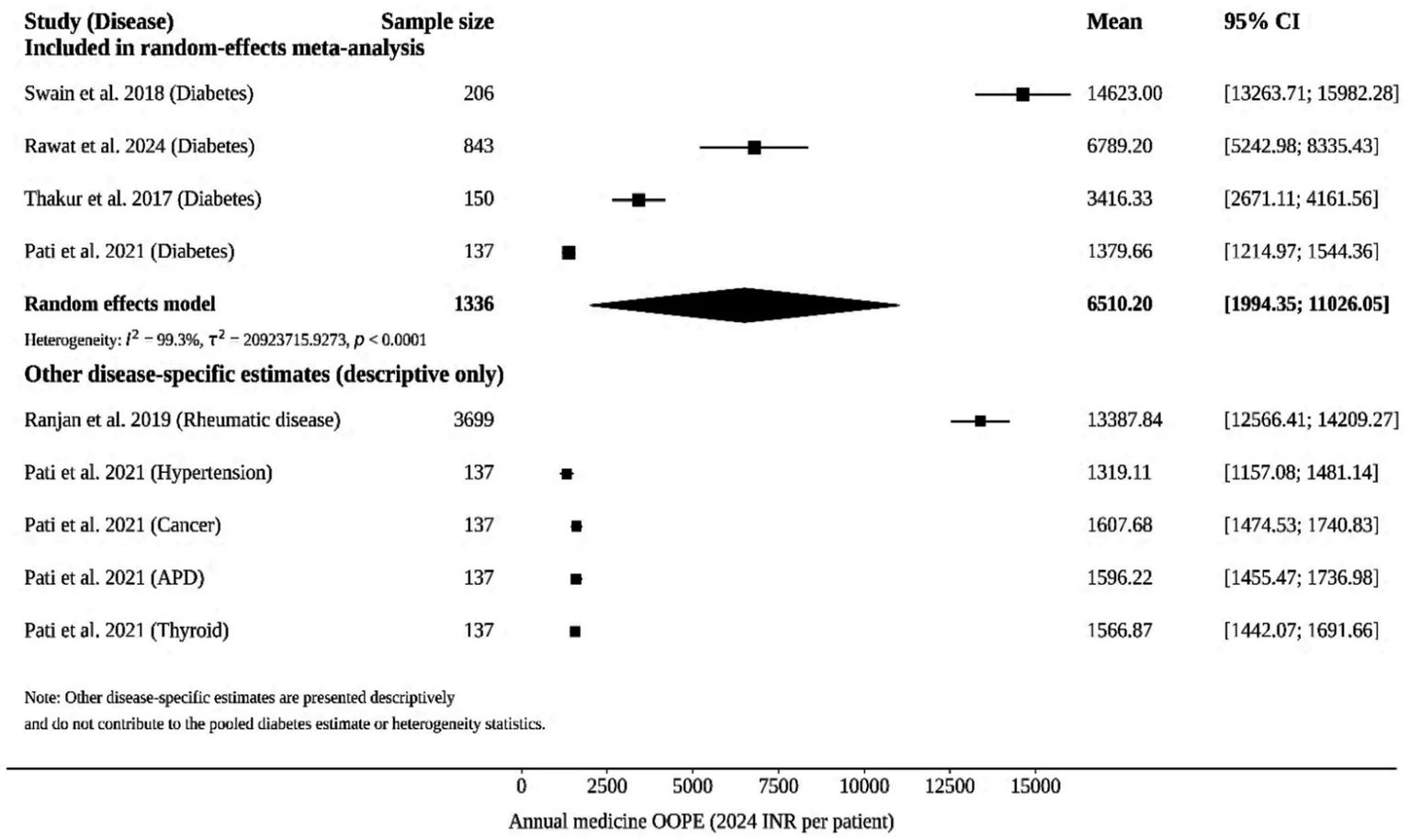
Disease-specific variation in annual medicine-related out-of-pocket expenditure (OOPE) among patients with chronic diseases in India, with costs adjusted to constant 2024 Indian Rupees (INR). Four independent estimates of medicine-related OOPE among patients with diabetes were quantitatively synthesized using a random-effects model. Estimates for rheumatic disease, hypertension, cancer, acid peptic disease, and thyroid disorders were presented descriptively because of limited evidence and/or non-independent estimates derived from the same study population. Squares represent study-specific mean estimates with 95% confidence intervals (CIs), and the diamond represents the pooled random-effects estimate for diabetes.

The findings of the subgroup and descriptive analyses are summarised in Figures 4–6. Subgroup analyses examined variation in medicine-related OOPE between chronic and non-chronic conditions and between public and private healthcare settings, while disease-specific estimates and inpatient, outpatient patterns were additionally examined descriptively where formal pooling was not appropriate.

The majority of the studies provided data on both rural and urban populations (23 studies; 76.7%). Five studies (16.7%) were conducted exclusively in urban areas, and one study (3.3%) was conducted solely among the rural population. One study (3.3%) did not specify the rural or urban setting, suggesting a broader coverage of mixed rural and urban environments.

Several studies focused on sex-specific populations, while some did not disaggregate data by sex. Nine studies (30.0%) reported female-specific participants, six studies (20.0%) male-specific participants, and 13 studies (43.3%) included both male and female participants. Two studies (6.7%) did not provide sex-specific information.

The subgroup analysis comparing chronic and non-chronic conditions (Figure 4) revealed that Medicine-related OOPE was higher among patients with chronic conditions than among those with non-chronic conditions; however, the difference between the subgroups was not statistically significant. The studies included in this review suggest that OOPE on medicine among populations with chronic diseases accounted for over 30 to 80% of overall OOPE on healthcare. This demonstrates a substantial cost burden of medicines for chronically ill patients.

The healthcare sector analysis (Figure 5) showed that the pooled mean medicine-related OOPE was numerically higher in private healthcare facilities than in public healthcare facilities; however, the difference between the two settings was not statistically significant. Research has shown that in the public healthcare sector, approximately 30 to 45 per cent of total OOPE is attributed to medicines, with a similar proportion observed in the private sector, differences in medicine-related expenditure have been reported between the two sectors.

The disease-specific analysis (Figure 6) demonstrated considerable variation in annual medicine-related OOPE across chronic disease populations. Four independent estimates of medicine-related OOPE among patients with diabetes were quantitatively pooled using a random-effects model, while estimates for rheumatic disease, hypertension, cancer, acid peptic disease, and thyroid disorders were presented descriptively because of limited evidence and/or non-independent estimates derived from the same study population. Multiple estimates derived from the same study population were not treated as independent observations. Medicine expenditure shares were reported to be high in studies focusing on diabetes (eight studies; 26.7%), hypertension (five studies; 16.7%), and cancer (four studies; 13.3%). Five studies (16.7%) considered both diabetes and hypertension, while four studies (13.3%) examined spending on chronic diseases without specifying particular conditions. Two studies (6.7%) involved mixed acute and chronic conditions, while one study each (3.3%) focused on acute conditions, joint pain, and lymphatic filariasis.

The descriptive comparison of inpatient and outpatient care indicated differences in the proportion of medicine-related expenditure. For inpatient care, medicines accounted for approximately 30–50% of total healthcare OOPE, whereas for outpatient care, the proportion was higher, ranging from approximately 60–80%. These estimates were analysed separately because of differences in outcome units and should therefore be interpreted as descriptive patterns rather than as a formal statistical comparison between inpatient and outpatient care.

Other studies have also indicated the broader financial implications of high OOPE in medicine. Medicines were identified as a significant source of CHE in several studies, with a high proportion of CHE observed in households affected by diabetes. In one study, the cost of medicines alone was reported to push approximately 38 million people into poverty (8), emphasising the severe economic impact of medicine expenses on individuals.

These results are presented with caution because the included studies varied in design, population, and outcome measurement. However, findings across the included studies indicate that medicines constitute a substantial component of OOPE, particularly among patients with chronic diseases and those receiving outpatient care.

### OOPE on medicine incurred by chronic patients

**Figure 7:**
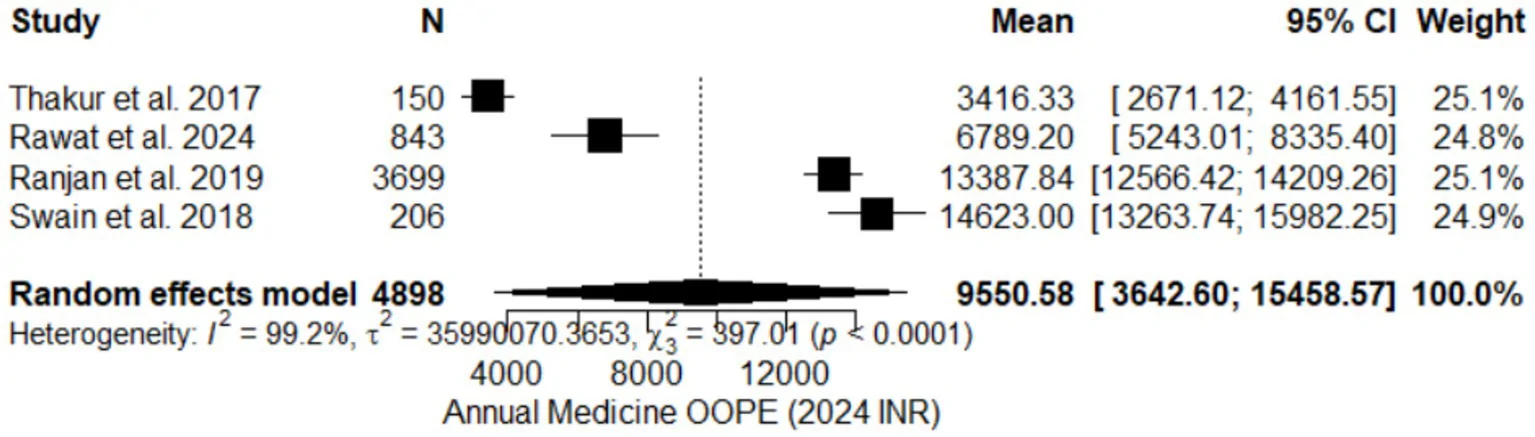
Random-effects forest plot of the pooled mean annual out-of-pocket expenditure (OOPE) on medicines among patients with chronic diseases in India based on the available evidence, with costs adjusted to 2024 Indian Rupees (INR) using the Consumer Price Index (CPI). Squares represent study-specific mean estimates with 95% confidence intervals (CIs), and the diamond represents the pooled random-effects estimate.

Figure 7 presents the random-effects meta-analysis of the mean annual out-of-pocket expenditure (OOPE) on medicines among patients with chronic diseases in India based on the available evidence. Four studies comprising 4,898 participants were included in the quantitative synthesis. The pooled mean annual medicine OOPE was ₹9,550.58 per patient (95% CI: ₹3,642.60–₹15,458.57). Between-study heterogeneity was extremely high (*I*^2^ = 99.2%, τ^2^ = 35,990,070.37; Cochran’s Q = 397.01, *p* < 0.0001), indicating substantial variability in expenditure estimates across the included studies. Given this high level of heterogeneity, a random-effects model was considered appropriate to account for between-study variability.

The wide confidence interval around the pooled estimate further reflects the considerable uncertainty arising from differences in disease profiles, healthcare settings, patient populations, and expenditure patterns among the included studies. Consequently, the pooled estimate should be interpreted as the average annual medicine OOPE across heterogeneous chronic disease populations, rather than as a single universal value applicable to all chronic disease patients in India.

### OOPE on medicines as part of total OOPE on healthcare

**Figure 8:**
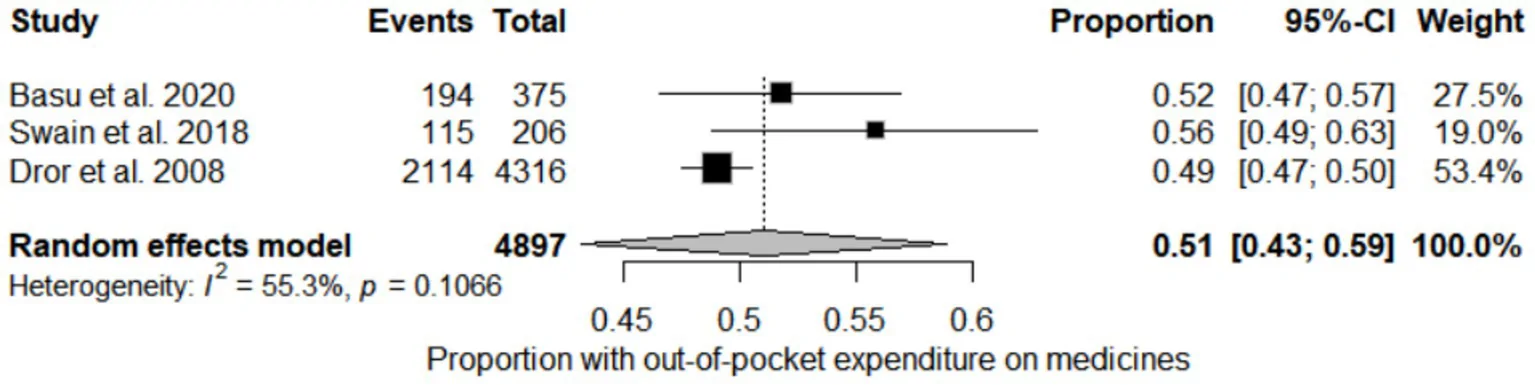
Pooled proportion of individuals incurring OOPE on medicines, with study-specific estimates and 95% confidence intervals shown under a random-effects model using a forest plot.

Figure 8 shows the pooled proportion of individuals incurring OOPE on medicines in India. Across the included studies, the proportion of individuals reporting OOPE on medicines ranged from 49 to 56%. The random-effects meta-analysis estimated that 51% of individuals incurred out-of-pocket expenditure on medicines (pooled proportion: 0.51; 95% CI: 0.43–0.59). This estimate represents the proportion of individuals reporting medicine-related OOPE in the included studies and should not be interpreted as indicating that the remaining individuals had their medicine costs covered by insurance or government schemes, as these reasons were not consistently reported across studies.

### Chronic vs. non-chronic patients

Figure 4 presents the subgroup random-effects meta-analysis comparing annual medicine OOPE between patients with chronic and non-chronic conditions in India based on available evidence. The pooled mean annual medicine OOPE among patients with chronic conditions was ₹9,550.58 per patient (95% CI: ₹3,642.60–₹15,458.57) based on four studies comprising 4,898 participants. In comparison, the pooled mean annual medicine OOPE among patients with non-chronic conditions was ₹4,383.79 per patient (95% CI: ₹2,722.47–₹6,045.10) based on four studies involving 3,627 participants.

Substantial between-study heterogeneity was observed in both subgroups, with *I*^2^ = 99.2% for chronic conditions and *I*^2^ = 99.3% for non-chronic conditions, indicating considerable variability in expenditure estimates across the included studies. Consequently, random-effects models were used to account for this heterogeneity.

Although the pooled mean medicine OOPE was approximately 2.2 times higher among patients with chronic conditions than among those with non-chronic conditions, the test for subgroup differences was not statistically significant (*χ*^2^ = 2.72, df = 1, *p* = 0.0989). This indicates that, based on the available evidence, there is insufficient statistical evidence to conclude that the pooled mean annual medicine OOPE differs between chronic and non-chronic conditions, despite the higher point estimate observed for chronic diseases.

### Public vs. private

Figure 5 presents the subgroup random-effects meta-analysis of medicine out-of-pocket expenditure (OOPE) per inpatient episode according to healthcare setting (public versus private) in India, with all costs adjusted to 2024 Indian Rupees (INR). In the public healthcare setting, the pooled mean medicine OOPE was ₹3,271.20 per inpatient episode (95% CI: ₹852.31–₹5,690.10) based on two studies involving 1,433 participants. In the private healthcare setting, the pooled mean medicine OOPE was ₹4,135.81 per inpatient episode (95% CI: −₹2,244.42 to ₹10,516.04) based on two studies comprising 1,841 participants.

Substantial between-study heterogeneity was observed in both subgroups, with *I*^2^ = 95.7% for public healthcare settings and *I*^2^ = 99.2% for private healthcare settings, indicating considerable variability in medicine expenditure estimates across the included studies. Consequently, random-effects models were applied to account for the observed heterogeneity.

Although the pooled estimate was numerically higher for the private healthcare setting than for the public healthcare setting, the test for subgroup differences was not statistically significant (*χ*^2^ = 0.06, df = 1, *p* = 0.8039). Therefore, based on the available evidence, there was no statistically significant difference in medicine OOPE per inpatient episode between public and private healthcare settings.

### Disease-specific variation in medicine-related OOPE

Figure 6 presents disease-specific variation in annual medicine-related OOPE among patients with chronic diseases in India. Four independent estimates reporting medicine-related OOPE among patients with diabetes were quantitatively synthesized using a random-effects model. The annual medicine-related OOPE among diabetes patients ranged from ₹1,379.66 to ₹14,623.00 per patient, with a pooled mean of ₹6,510.20 per patient (95% CI: ₹1,994.35–₹11,026.05).

Substantial between-study heterogeneity was observed among the diabetes estimates (*I*^2^ = 99.3%; τ^2^ = 20,923,715.93; Cochran’s Q = 423.74, *p* < 0.001), indicating considerable variation in medicine-related OOPE across the included diabetes populations. This variability may reflect differences in study settings, populations, healthcare utilization, treatment requirements, geographical context, and methods of expenditure measurement.

Disease-specific estimates were analysed quantitatively for diabetes, while estimates for other chronic diseases were presented descriptively because of limited evidence and/or non-independent observations. Ranjan et al. reported annual medicine-related OOPE of ₹13,387.84 for rheumatic disease (53), while Pati et al. reported estimates of ₹1,319.11 for hypertension, ₹1,607.68 for cancer, ₹1,379.66 for diabetes, ₹1,596.22 for acid peptic disease, and ₹1,566.87 for thyroid disorders. The disease-specific estimates reported by Pati et al. were derived from the same study population (*n* = 137); therefore, the estimates for hypertension, cancer, acid peptic disease, and thyroid disorders were not treated as independent observations in quantitative meta-analysis, while the diabetes estimate was included in the prespecified diabetes-specific synthesis.

Overall, Figure 6 demonstrates substantial variation in medicine-related OOPE across chronic disease categories, while the quantitative synthesis provides a pooled estimate specifically for diabetes. Given the very high heterogeneity among the diabetes studies, the pooled estimate should be interpreted as a summary of the available evidence rather than as a precise national estimate.

## Discussion

In this systematic review and meta-analysis, we synthesised evidence on the burden of medicine-related OOPE among patients with chronic conditions in India and provided a comprehensive assessment of expenditure patterns across disease categories, healthcare settings, and healthcare facilities. To our knowledge, this is the first systematic review and meta-analysis to consolidate evidence specifically on medicine-related OOPE in India and to examine variation across disease categories, healthcare sectors, and care settings through subgroup and descriptive analyses. Overall, five key findings emerge from our review. Although the review included a range of chronic conditions, including diabetes, hypertension, cancer, thyroid disorders, rheumatic disease, APD, and studies of chronic diseases without disease-specific classification, the evidence base was dominated by diabetes and hypertension. Therefore, the findings should be interpreted with greater confidence for these conditions than for less frequently represented chronic diseases.

First, our findings show that medicine-related OOPE constitutes a substantial component of total healthcare OOPE among patients with chronic conditions in India (9, 17). Considerable variation in annual medicine-related OOPE was observed across the included studies, reflecting differences in disease conditions, study populations, healthcare settings, and expenditure patterns (18). Overall, individuals with chronic conditions incurred higher medicine-related OOPE than those with non-chronic conditions; however, the pooled subgroup difference was not statistically significant.

The observed heterogeneity (*I*^2^ = 99.2%) suggests that medicine-related OOPE differs substantially across studies, likely reflecting variations in disease type, prescribing practices, healthcare utilization, regional differences, medicine prices, and study methodologies (33). Therefore, although the pooled estimate provides a useful summary measure, it should be interpreted cautiously because it represents the average of highly heterogeneous populations rather than a common underlying expenditure value.

Across the included studies, medicines accounted for a substantial proportion of total healthcare OOPE, although the magnitude varied by healthcare setting. Medicine-related expenditure generally accounted for approximately 30–50% of total OOPE during inpatient care and 60–80% during outpatient care (19, 20). This highlights the key role of medicines as the primary cause of the financial burden associated with healthcare for patients with chronic conditions. The magnitude of OOPE related to medicine expenditure indicates that the current financial protection mechanisms in India are inadequate for the long-term pharmacological management of chronic diseases (21, 30, 49). Previous evidence has also demonstrated the broader financial consequences of medicine expenditure, with an estimated 38 million people in India reported to have been pushed into poverty because of out-of-pocket spending on medicines.

Approximately half of the included participants incurred medicine-related out-of-pocket expenditure. This finding should not be interpreted as indicating that the remaining participants were fully protected by health insurance or government health schemes. Rather, the absence of reported medicine-related OOPE may reflect several factors, including receipt of free medicines through public health facilities, enrolment in government medicine programmes, differences in healthcare-seeking behaviour, variation in study populations, differences in reporting periods, or study-specific definitions of medicine expenditure. Because the included studies did not consistently report the reasons for the absence of medicine-related OOPE, no conclusions regarding insurance coverage or financial protection can be drawn.

The findings of this study provide valuable insights about the state of Universal Health Coverage (UHC) in India. Schemes run by the Government of India such as the Ayushman Bharat Scheme, launched in 2018 and expected to be expanded further by 2026, have increased population coverage to around 61 crore people, representing nearly 45% population of India (22, 23). Although coverage for inpatient hospitalization has increased, outpatient medicine costs for patients requiring regular follow-up and long-term treatment remain largely uncovered. This leads to households with chronic patients relying heavily on direct out-of-pocket payments for the purchase of their medicines. The limited coverage for chronic patients for the purchase of medicine may partly explain the persistently high OOPE on medicine by chronic patients (29, 52).

Chronic patients that visit private healthcare settings for outpatient consultation tend to purchase branded medicine which reflects a broader purchase behaviour amongst patients (48). These patients tend to believe in the efficacy of branded medicine, the comfort of a steady supply chain and easy accessibility of branded medicine as they are available in local pharmacies. In contrast, public sector medicine procurement and free drug distribution programmes are run by various state governments across India to reduce the burden of OOPE on medicine but uneven availability of medicines restricts the impact of these schemes.

The Pradhan Mantri Bhartiya Janaushadhi Pariyojana (PMBJP) scheme run by the Government of India with the aim to provide affordable generic medicines to patients across India but faces issues of limited access, limited availability of medicine and limited awareness amongst patient about the scheme (24).

Nevertheless, the findings of this review suggest that substantial gaps in medicine affordability and financial protection remain, particularly among socioeconomically disadvantaged populations and individuals requiring long-term chronic disease management. Strengthening public drug procurement systems, improving the availability of essential medicines in government facilities, and expanding insurance coverage for outpatient medicines may therefore be important strategies for reducing healthcare financing inequality and catastrophic health expenditure associated with chronic diseases in India (32, 44, 47).

Our second key observation is that several individual studies reported higher medicine-related OOPE among patients with chronic conditions than among those with non-chronic (acute) conditions. Monthly household medicine expenditure among chronic patients was approximately ₹1,012, compared with ₹302 for non-chronic patients (21). This trend was persistently reported in multiple studies, which found higher medicine-related OOPE among chronic patients and relatively lower expenditure among those with non-chronic conditions. Previous research has also identified reduced medicine-related OOPE among non-chronic conditions, emphasising that the management of chronic diseases is associated with a substantially greater financial burden (25, 26).

Our subgroup analysis comparing chronic and non-chronic conditions found that the pooled mean annual medicine-related OOPE was numerically higher among patients with chronic conditions (₹9,550.58 per patient) than among those with non-chronic conditions (₹4,383.79 per patient). However, the subgroup difference was not statistically significant (*p* = 0.0989), indicating insufficient statistical evidence to conclude that annual medicine-related OOPE differed between the two groups. However, the subgroup difference was not statistically significant, suggesting that the available evidence does not support a meaningful difference in annual medicine-related OOPE between the two groups. Both subgroups exhibited substantial between-study heterogeneity (*I*^2^ = 99.3%), indicating considerable variability in expenditure estimates across studies. This heterogeneity is likely attributable to differences in disease characteristics, healthcare settings, patient populations, treatment regimens, and methods used to estimate medicine costs.

The absence of a statistically significant difference should be interpreted with caution. The chronic subgroup included a broad range of long-term conditions such as diabetes, rheumatic disease, hypertension, cancer, thyroid disorders, and acid peptic disease, whereas the non-chronic subgroup First, our findings show that OOPE on medicines constitutes the largest component of total healthcare OOPE among patients with chronic conditions in India comprised studies of heterogeneous conditions, including enteric fever, general inpatient populations, and cost-of-illness estimates (41). These differences in clinical conditions and study designs may have contributed to the substantial heterogeneity observed within both subgroups. Consequently, the pooled estimates should be considered summary measures rather than definitive comparisons of medicine-related OOPE between chronic and non-chronic conditions.

Our third key finding is that the pooled mean medicine-related OOPE was numerically higher in private healthcare facilities than in public healthcare facilities; however, the difference between the two settings was not statistically significant (*p* = 0.8039). Therefore, this finding should be interpreted cautiously, particularly given the substantial between-study heterogeneity and the limited number of studies contributing to each subgroup. The observed tendency toward higher medicine-related OOPE in private facilities may reflect several structural and systemic factors. Private providers may have greater use of branded medicines and greater reliance on fee-for-service models, while patients accessing private facilities may also be more dependent on private pharmacies and less able to benefit from publicly subsidised medicine supply systems (46). These factors may contribute to higher medicine expenditure in private settings, although the available evidence does not establish a statistically significant difference between public and private facilities.

However, this finding should be interpreted with caution because the subgroup analysis comparing public and private healthcare settings was based on only two studies in each subgroup. The substantial between-study heterogeneity observed within both subgroups resulted in imprecise pooled estimates and wide confidence intervals. Therefore, the comparison between public and private healthcare settings should be considered exploratory and requires confirmation from additional high-quality studies using standardized methods for estimating medicine-related out-of-pocket expenditure on medicines.

Our fourth key finding relates to the proportion of medicines within total OOPE across healthcare settings. In the studies included, medicines consistently accounted for a substantial share of overall healthcare OOPE, although the extent of this proportion varied by care setting. Generally, the percentage of medicine-related OOPE was lower for inpatient care and higher for outpatient care.

Several studies have found that medicines account for approximately 19–47% of inpatient OOPE and 59–86% of outpatient OOPE (43). For instance, some studies reported that around 30–43% of inpatients incurred medicine-related OOPE, whereas 62–66% of outpatients incurred OOPE. Other studies found that medicines accounted for approximately 53–54% of inpatient OOPE and 70–78% of outpatient OOPE, with similar results observed across all sectors. In some settings, medicines were the largest single contributor to OOPE, comprising up to 67–72% of total healthcare expenditure, and accounted for more than 50% of OOPE in a significant proportion of households.

Overall, despite considerable variation in the reported estimates, the available evidence indicates that medicines constitute a substantial share of total OOPE (50). The proportional contribution of medicine-related OOPE was generally higher in outpatient care than in inpatient care across the included studies. This descriptive pattern was observed across different healthcare sectors and population groups; however, the inpatient and outpatient estimates were not directly pooled because of differences in outcome units.

Our fifth key finding highlights substantial variation in medicine-related OOPE across chronic disease populations. Diabetes was the most frequently represented disease in the available quantitative evidence (39). Four independent diabetes estimates were quantitatively pooled, yielding a pooled mean annual medicine-related OOPE of ₹6,510.20 per patient (95% CI: ₹1,994.35–₹11,026.05). However, substantial heterogeneity was observed among the diabetes estimates (*I*^2^ = 99.3%), indicating considerable variation across study populations and settings.

Other disease-specific estimates for rheumatic disease, hypertension, cancer, acid peptic disease, and thyroid disorders were presented descriptively. Multiple disease-specific estimates reported by Pati et al. were derived from the same study population (*n* = 137) and were therefore not treated as independent observations across disease categories. The diabetes estimate from Pati et al. was included in the diabetes-specific quantitative synthesis, whereas the other disease-specific estimates from the same population were presented descriptively (42). Consequently, Figure 6 provides a quantitative pooled estimate for diabetes while illustrating the available descriptive evidence for other chronic disease categories.

These findings suggest substantial variation in medicine-related financial burden across chronic diseases while also highlighting important evidence gaps for several conditions. Given the very high heterogeneity among the diabetes studies, the pooled estimate should be interpreted cautiously and should not be considered a precise national estimate. Future research should generate standardized, disease-specific estimates of annual medicine-related OOPE across diverse settings to enable more reliable comparisons and inform targeted financial protection policies.

## Conclusion

The available evidence suggests that medicine-related out-of-pocket expenditure (OOPE) constitutes a substantial component of household healthcare spending in India, particularly among patients with chronic diseases who require continuous and long-term treatment. The findings highlight important gaps in financial protection and medicine affordability, especially within outpatient care and private healthcare settings where households remain heavily dependent on direct payments for essential medicines. Strengthening public-sector drug procurement and distribution systems, expanding insurance coverage for outpatient medicines, improving access to affordable generic medicines through initiatives such as Jan Aushadhi, and strengthening medicine price regulation may substantially reduce the financial burden associated with chronic disease management (51).

This review provides a comprehensive synthesis of evidence on medicine-related OOPE across disease categories, healthcare settings, and facility types in India. However, substantial methodological and contextual heterogeneity was observed across the included studies. Variability in disease profiles, healthcare utilisation patterns, recall periods, expenditure measurement methods, and regional healthcare contexts likely contributed to differences in pooled estimates. Therefore, pooled estimates should be interpreted as indicative summary patterns rather than precise national averages. Nevertheless, these variations also reflect the real-world diversity of healthcare utilisation and financing patterns across India. Despite these limitations, the consistency in the direction of findings across studies supports the broader conclusion that medicines represent a major and persistent source of healthcare-related financial burden in India.

## Limitation

The pooled estimate of annual medicine-related OOPE among patients with chronic diseases should be interpreted with caution because it was based on only four studies, with substantial between-study heterogeneity. A limitation of the subgroup analysis is that the non-chronic subgroup included studies with diverse disease conditions and healthcare contexts rather than a single homogeneous clinical category. Consequently, comparisons between chronic and non-chronic conditions should be interpreted cautiously, as differences in disease severity, treatment duration, and healthcare utilization may have influenced the pooled expenditure estimates.

## Research in context

### Evidence before this study

Out-of-pocket expenditure (OOPE) is the main source of healthcare financing in many low- and middle-income countries, including India. It is a major cause of financial burden for households. A large share of this spending is on medicines, especially for chronic diseases that require continuous and long-term treatment. Non-communicable diseases (NCDs) are a leading cause of death globally and are more common in low- and middle-income settings. Although many studies in India have examined patterns and determinants of overall OOPE, evidence specifically on medicine-related OOPE remains scattered and has not been systematically synthesised at the national level.

### Added value of this study

This study provides pooled estimates of medicine-related OOPE among Indian households managing chronic illness based on the available evidence. By combining evidence from 30 studies and over 1.6 million participants, it estimates the magnitude and variation of this burden. It also identifies differences by disease type, care setting, and provider sector.

### Implication of all the available evidences

Overall, out of pocket expenditure (OOPE) on medicines is a major driver of financial hardship in India. 7Medicine-related OOPE represents a substantial financial burden, particularly for patients requiring continuous and long-term treatment for chronic diseases. This burden varies across disease categories, outpatient and inpatient care, and both public and private healthcare sectors. The findings also show key evidence gaps and highlight the need for more regionally representative studies on medicine-related OOPE among chronic disease patients. This evidence is important for improving financial protection policies and supporting national economic development.

## Data Availability

The original contributions presented in the study are included in the article/Supplementary material, further inquiries can be directed to the corresponding author.
