# Supplementary material for: Out-of-pocket expenditure on medicine for chronic patients and its economic impact on Indian households: a systematic review and meta-analysis
Source: Front Med (Lausanne). 2026 Sep 4;13:1818895. doi: 10.3389/fmed.2026.1818895 (PMC13585513; doi:10.3389/fmed.2026.1818895)
Supplement: Supplementary file 1 [file Supplementary_file_1.DOCX]

**Out-of-pocket Expenditure on Medicine for Chronic Patients and its Economic Impact on Indian Households: A Systematic Review and Meta-Analysis**

**S1: Search Strategy**

A combination of keywords and Boolean operators will be used to identify relevant literature.

**Keywords:** “Out-of-Pocket Expenditure”, “Medicine”, “Drugs”, “Pharmaceutical Costs”, “Chronic Illness”

**Boolean Operators:
Example search string: PubMed**
("Out-of-Pocket Expenditure" OR "Expenditure" OR "Purchase") AND ("Medicine" OR "Drug" OR "Drugs") AND ("Chronic Illness" OR "Chronic Disease")

**Example search string: Scopus**

("Out-of-Pocket Expenditure" OR "Expenditure" OR "Purchase") AND ("Medicine" OR "Drug" OR "Drugs") AND ("Chronic Illness" OR "Chronic Disease")

**Example search string: Web of Sciences**

("Out-of-Pocket Expenditure" OR "Expenditure" OR "Purchase") AND ("Medicine" OR "Drug" OR "Drugs") AND ("Chronic Illness" OR "Chronic Disease")

**Filters:**

Country/Region: **India**

Language: **English**

Publication Date: **2008 to 2025**

**S2-A: Inclusion & Exclusion Criteria**

**Table 1**

| **Domain** | **Inclusion Criteria** | **Exclusion Criteria** |
| --- | --- | --- |
| **Study setting** | Studies conducted in India | Studies conducted outside India |
| **Population** | Individuals or households incurring out-of-pocket expenditure (OOPE) on medicines | Studies limited to providers, facilities, or system-level costs without patient- or household-level data |
| **Disease scope** | Studies involving chronic diseases, non-chronic (acute) diseases, or both together | Studies unrelated to disease-specific healthcare expenditure |
| **Exposure (KEY CRITERION)** | Medicine-specific OOPE, defined as direct payments by individuals or households for medicines not reimbursed by insurance or government schemes | Studies reporting only total health expenditure or total OOPE, where medicine-related OOPE cannot be isolated or extracted |
| **Health expenditure reporting** | Studies where medicine-related OOPE is reported separately, as a proportion of total OOPE, or can be clearly identified/extracted | Studies reporting only overall healthcare, hospitalization, consultation, diagnostic, or transport costs without identifiable medicine components |
| **Outcomes   quantitative** | Mean, median, total, proportion, or share of OOPE on medicines | Studies reporting only clinical outcomes or service utilization |
| **Outcomes   qualitative** | Descriptive or narrative evidence on the magnitude, drivers, distribution, or economic impact of medicine-related OOPE | Studies with no quantitative or qualitative information on medicine-related OOPE |
| **Healthcare setting** | Inpatient and/or outpatient care | N/A |
| **Provider type** | Public and/or private healthcare facilities | N/A |
| **Study design** | Quantitative, mixed-methods, or descriptive observational studies (cross-sectional, cohort, facility-based surveys, secondary analyses) | Editorials, commentaries, letters, conference abstracts without full text |
| **Data availability** | Extractable numerical data (for meta-analysis) or relevant descriptive data (for qualitative synthesis) | Insufficient data for both quantitative and qualitative synthesis |
| **Publication type** | Peer-reviewed journal articles only | Government or institutional reports; reviews, systematic reviews, and meta-analyses (references screened only) |
| **Duplicates** | The most complete or most recent study retained | Duplicate publications using the same dataset |
| **Time period** | Published between January 1, 2008, and March 31, 2025 | Published outside the specified period |
| **Language** | English | Non-English articles without a reliable translation |

**S2-B: Research Questions**

1. What is the magnitude of household OOP spending on medicines among chronically ill patients in India?
2. How is OOPE allocated between chronic and non-chronic disease conditions?
3. How does the burden of household OOPE on medicines vary by healthcare facility type and care setting in India?
4. How does household out-of-pocket spending on medicines vary across different chronic diseases in India?

**S2-C: Data extraction**

**A. Study Identification and Design**

- Author(s)
- Year of publication
- Study period
- Journal
- Study design (cross-sectional / prospective / longitudinal / qualitative)
- Geographic coverage (national / state / regional)
- Region of India (North / South / Central / West / Mixed)

**B. Study Population Characteristics**

- Total sample size
- Unit of analysis (individual / household)
- Population type (general population / chronic disease population)
- Age group(s)
- Sex distribution (if reported)
- Rural / urban setting

**C. Disease Classification**

- Chronic
- Non-chronic (acute)
- Mixed / unspecified

Specific diseases reported (e.g., diabetes, cardiovascular disease, cancer, respiratory disease, multimorbidity)

**D. Out-of-Pocket (OOP) Expenditure on Medicines – Core Outcome**

- Mean OOP medicine expenditure
- Median OOP medicine expenditure (if reported)
- Measure of dispersion (SD / IQR / CI)
- Time reference (annual / monthly / per episode)
- Currency reported
- Currency converted to INR
- Data eligible for meta-analysis (Yes / No)

**E. Chronic vs Non-Chronic Disease Comparison**

Extracted where explicitly reported

- OOP medicine expenditure for chronic conditions
- OOP medicine expenditure for non-chronic conditions
- Effect measure mean
- Sample size per subgroup

**F. Disease-Specific OOP Medicine Expenditure**

Where available

- Disease category
- Mean / median OOP medicine expenditure
- Time period
- Sample size per disease

**G. Healthcare Facility Type and Care Setting**

Care setting

- Outpatient
- Inpatient

Facility type

- Public
- Private

OOP medicine expenditure by

- Inpatient vs outpatient care
- Public vs private facilities

**H. OOP Medicines as a Share of Total OOP**

- Proportion of households incurring OOP on medicines
- Share (%) of medicines in total OOP health expenditure
- Measure of uncertainty (if reported)

**I. Socio-Demographic and Economic Variables**

- Extracted only when quantitatively reported
- Age-group–wise OOP medicine expenditure
- Wealth quintile / economic status
- Rural vs urban differences

Catastrophic health expenditure related to medicines

**S3: PRISMA 2020 flow diagram for new systematic reviews which included searches of databases and registers only**

Studies included in systematic review, (n = 30)

**Identification**

Records obtained

(n = 206)

PubMed = 29

Scopus = 127

WOB = 50

Duplicate exclude (n = 63)

Records screened by title and abstract n= 143

Records excluded title and abstract	(n=50)

Full-text articles assessed for eligibility(n=93)

Not have full access

(n=18)

n= 18

Articles remaining (n=75)

Article did not meet research objective (n =41)

Articles remaining (n=34)

**Identification of studies via databases and registers**

**Screening**

**Included**

Reports of included meta– analysis (n =11)

Article did not match Research Question (n=4)

Fig. 2. PRISMA flow diagram of study selection for the systematic review and meta-analysis on household Out-of-Pocket expenditures on medicines in India.mm

**S4: Fig 1 Map shows the Geographical Distribution of studies included in the review**

**s**

**S5-A - Appendix Table 2 — Characteristics of included studies**

| **Author** | **Year** | **Study design** | **Region** | **Area**  (Rural Urban & Both) | **Population Type** | **Age Group** | **Sex** | **Sample size** | **Disease Categories** | **Specific**  **Categories** | **Facility type**  **(Public, Private& Both)** | **Care setting**  **(Inpatients, Outpatients& Both)** |
| --- | --- | --- | --- | --- | --- | --- | --- | --- | --- | --- | --- | --- |
| Goswami & Anand | 2024 | cross-sectional | Mixed | Both | General population | 45–59, 60–74, ≥75 | 54% female | 65,562 individuals (8,429 diabetics) | Both | Diabetes; hypertension; cancer; CVD; stroke; chronic lung disease | Both | Inpatient |
| Tripathy et al. | 2016 | cross-sectional | Mixed | Both | Chronic disease | 49 | 26% female | 14,609 NCD hospitalisation episodes | Chronic | Cancer; CVD; respiratory; endocrine; injuries | Both | Inpatient |
| Ranjan et al. | 2019 | Mixed-methods (cross-sectional + qualitative interviews) | Mixed | Both | Chronic disease | All ages | 50.4% female (IP); 65.2% female (OP) | 65,932 households; 333,104 individuals | Chronic | Rheumatic diseases (joint/bone pain, back pain, arthritis) | Both | Both |
| Rajasulochana et al. | 2023 | cross-sectional | Mixed | Both | Chronic disease | ≥35 years | 64% female (IP); 56% female (OP) | 1,351 IP episodes; 6,379 OP visits | Chronic | Hypertension | Both | Both |
| Prinja et al. | 2012 | cross-sectional | North | Both | General population | All ages | Not reported | 3,305 households (states combined) | Mixed | All causes of illness | Both | Both |
| Tripathy & Prasad | 2020 | cross-sectional | Mixed | Both | Chronic disease | All ages (majority ≥45 years) | 84% male (IP); 67% male (OP) | 333,104 individuals; 38 IP episodes; 36 OP episodes (LF) | Chronic | Lymphatic filariasis | Both | Both |
| Koul et al. | 2019 | Prospective observational study | North | Not reported | Chronic disease | age 68 years | 54% male | 129 patients with severe AECOPD | Chronic | Severe COPD (acute exacerbations) | Public (tertiary hospital) | Inpatient |
| Bose & Dutta | 2018 | cross-sectional | Mixed | Both | General population | All ages | Not reported | ≈15,000 households across 3 states | Mixed | All causes (communicable & non-communicable) | Both | Inpatient |
| Prinja et al. | 2023 | cross-sectional | Mixed | Both | Chronic disease | All ages | ≈58% female | 12,148 cancer patients | Chronic | Cancer (multiple sites) | Both | Both |
| Jacob et al. | 2023 | cross-sectional | South | Urban (low-resource setting) | Chronic disease | ≥18 years | 72.6% female | 230 individuals with diabetes (from 1065 households) | Chronic | Diabetes mellitus | Private facilities only | Outpatient (primarily) |
| Pati et al. | 2021 | Quasi-randomized cluster trial | South | Rural | Chronic disease | Adults with diabetes and/or hypertension | both | 1,154 patients (baseline); 1,252 (endline) across 39 PHCs | Chronic | Diabetes; Hypertension | Public (PHCs) | Outpatient (primary care) |
| Dehury et al. | 2025 | cross-sectional | East | Both | General population (acute &chronic illness) | ≥18 years | 60.2% male | 902 households (173 with medicine OOP) | Mixed | Acute illness; Chronic illness (hypertension, diabetes, arthritis, etc.) | Both | Outpatient (predominantly) |
| Bhehra& Pradhan | 2021 | cross-sectional | Mixed | Both | Chronic disease | Adults ≥45 years | both | ≈72,000 households | Chronic | Hypertension; Diabetes; Heart disease; Cancer; Multimorbidity | Both | Both |
| Singh et al. | 2022 | cross-sectional | North | Urban | General population (acute & chronic) | All ages | both | 3,270 urban poor households | Mixed | Acute illness; Chronic illness | Both | Both |
| Singh et al. | 2016 | cross-sectional | North | Both | General population (acute & chronic illness) | All ages | 55% male | 1,010 households (4,227 individuals) | Mixed | Acute and chronic illnesses | Both | Both |
| Pati et al. | 2014 | cross-sectional | Mixed | Both | Chronic disease | ≥18 years | ≈50% female | 10,973 individuals | Chronic | Hypertension; Diabetes; CVD; Asthma; Arthritis; Stroke; Multimorbidity | Both | Both |
| Swain et al. | 2018 | cross-sectional | East | Both | Chronic disease | ≥25 years | 67% male | 206 patients with type 2 diabetes | Chronic | Type 2 Diabetes Mellitus | Public (tertiary hospital) | Outpatient |
| Sharma et al. | 2017 | cross-sectional | North | Both | General population (acute & chronic hospitalization) | All ages | 55.1% male | 79,743 households (13,253 hospitalized individuals) | Mixed | Communicable; NCDs; injuries | Both | Inpatient |
| Ambade et al. | 2022 | cross-sectional | Mixed | Both | General population | All ages | 64.3% female (inpatients) | 43,781 inpatients; 8,914 outpatients | Mixed | All diseases | Both | Both |
| Rout & Choudhury | 2018 | cross-sectional | East | Both | General population (hospitalised) | All ages | ≈60% female | 239 inpatients | Mixed | Surgical; Non-surgical; Childbirth | Public (secondary level) | Inpatient |
| George et al. | 2024 | Secondary data analysis | Mixed | Both | Chronic disease | All ages | Both | NSS cancer cases (national sample) | Chronic | Cancer | Both | Both |
| Shweta & Nanda | 2019 | cross-sectional | Mixed | Both | Chronic disease | ≥18years | Both | 65,932 households (elderly subsample) | Chronic | Diabetes mellitus | Both | Both |
| Bose & Banerjee | 2019 | cross-sectional | Mixed | Both | Chronic disease | ≥60 years | Both | 65,932 households; 333,104 individuals | Chronic | NCDs (hypertension, diabetes, CVD, etc.) | Both | Both |
| Selvaraj S; Farooqui HH; Karan A | 2018 | cross-sectional | Mixed | Both | General population (chronic & non-chronic) | All ages | Both | ≈100,000–125,000 households per round; HMS ~72,000 | Mixed | Communicable, NCDs, injuries, cancer | Both | Both |
| Shahrawat R; Rao KD | 2011 | cross-sectional | Mixed | Both | General population | All ages | Both | 124,644 households | Mixed | All conditions | Both | Both |
| Dror et al. | 2008 | cross-sectional | Mixed | Both | General population (acute & chronic) | All ages | Both | ≈3,700 illness episodes | Mixed | Acute illnesses; Chronic illnesses (asthma, CVD, diabetes, etc.) | Both | Both |
| Thakur et al. | 2017 | cross-sectional | North | Urban | Chronic disease | ≥30 years | Both | 400 adults with diabetes | Chronic | Diabetes mellitus | Both | Both |
| Basu et al. | 2020 | cross-sectional | North India | Urban | Chronic disease | Adults (≥18 years) | Both sexes | 375 | NCD | Type 2 diabetes mellitus | Public tertiary hospital | Outpatient |
| Kumar et al. | 2021 | cross-sectional | Mixed | Mixed | Hospitalized patients | Children and adults | Both | 1275 | Infectious diseases | Enteric fever | Mixed (Tier 2 & Tier 3 hospitals) | Inpatient |
| Rawat et al. | 2024 | cross-sectional | North | Urban | Adults with diabetes | 35–65 years | Both | 843 | Non-communicable disease | Diabetes mellitus | Both | Outpatient / ambulatory |

**S5-B: Appendix Table 3-** Information on the measurement and reporting of medicine-related OOP

| **Author** | **year** | **Time reference** | **Recall period** | **Currency reported** | **Converted to INR (Yes/No)** | **OOPE measure reported** |
| --- | --- | --- | --- | --- | --- | --- |
| Goswami & Anand | 2024 | Model-based | 12 months (hospitalisation) | INR | INR | Mixed |
| Tripathy et al. | 2016 | Per episode | 365 days | USD | Not converted | Proportion |
| Ranjan et al. | 2019 | Per hospitalization episode | 15 days (OP); 365 days (IP) | INR | INR | Mean |
| Rajasulochana et al. | 2023 | Per episode | 365 days (IP); 15 days (OP) | INR | INR | Mean |
| Prinja et al. | 2012 | Per episode per visit | 365 days (IP); 15 days (OP) | INR | INR | Proportion |
| Tripathy & Prasad | 2020 | Per episode | 365 days (IP); 15 days (OP) | USD | Converted by authors | Median |
| Koul et al. | 2019 | Per hospitalization episode | Current episode (hospital stay + 2-week follow-up) | INR | INR | Median |
| Bose & Dutta | 2018 | Per hospitalization episode | 365 days (hospitalization) | INR | INR | Mean |
| Prinja et al. | 2023 | Per visit / per episode / annual | OP visit / IP episode / annual | INR | INR | Proportion |
| Jacob et al. | 2023 | Annual | Annual (12-month recall) | INR | INR | Median |
| Pati et al. | 2021 | Change from baseline to endline | Baseline–endline (18-month intervention) | INR | INR | Mean |
| Dehury et al. | 2025 | Monthly | Last 4 weeks (monthly) | INR | INR | Proportion |
| Bhehra& Pradhan | 2021 | Annual | Annual | INR | INR | Proportion |
| Singh et al. | 2022 | Annual | 15 days (OP); 365 days (IP) | INR | INR | Mean |
| Singh et al. | 2016 | Monthly | 30 days (OP); 365 days (IP) | INR | INR | Mean |
| Pati et al. | 2014 | Per visit | Last OP visit (12 months); last IP visit (3 years) | INR | INR | Mean |
| Swain et al. | 2018 | Monthly | Monthly | INR | INR | Mean |
| Sharma et al. | 2017 | Per hospitalization episode | Last 365 days (hospitalization) | INR | INR | Mean |
| Ambade et al. | 2022 | Annual | Annual | INR | INR | Proportion |
| Rout & Choudhury | 2018 | Per episode | Per hospitalization episode | INR | INR | Mean |
| George et al. | 2024 | Annual (IP); 15-day (OP) | Annual (IP); 15 days (OP) | INR | INR | Mixed |
| Mohanty et al. | 2018 | OP & IP reference | 15 days (OP); 365 days (IP) | INR | INR | Proportion |
| Bose & Banerjee | 2019 | OP & IP reference | 15 days (OP); 365 days (IP) | INR | INR | Proportion |
| Selvaraj S; Farooqui HH; Karan A | 2018 | Monthly (converted) | 30 days (OP); 365 days (IP) | INR | INR | Proportion |
| Shahrawat R; Rao KD | 2011 | 30 days | 30 days (OP & IP) | INR | INR | Proportion |
| Dror et al. | 2008 | Per episode | Per illness episode | INR | INR | Mean |
| Thakur et al. | 2017 | Annual | Annual | INR | INR | Mean |
| Basu et al. | 2020 | Per month | 30 days | INR | INR | Proportion |
| Kumar et al. | 2021 | Per episode | Illness episode + 28 days | INR | INR | Mean |
| Rawat et al. | 2024 | Per year | Annual | INR | INR | Mean |

**S5-C: Appendix Table 4- Risk of bias**

| **Author (year) (reference** | **Region/ study area** | **Study design** | **Sample size(N)** | **Quality score** | **Quality status** |
| --- | --- | --- | --- | --- | --- |
| Selvaraj et al., 2018 | National | Cross-Sectional | 101,651 households in the 2011-12  65,932 households in the 2014 | 7 | High |
| Sharma et al. (2017) | Haryana | Cross-Sectional | 79,743 households, comprising 436,657 individuals  13,253 individuals (or episodes) | 8 | High |
| Swain et al. 2018 | Odisha | Cross-Sectional | 206 patients | 6 | moderate |
| Rawat et al. 2024 | Delhi | Cross-Sectional | 843 individuals with diabetes | 8 | High |
| Ranjan et al. 2019 | National | Cross-Sectional | 65,932 households / 333,104 individuals | 8 | High |
| Dehury et al. 2025 | Odisha | Cross-Sectional | 902 households | 8 | High |
| Dror et al. 2008 | National/Poor | Cross-Sectional | 3,531 households / 17,323 individuals / 4,316 illness episodes | 5 | Modrate |
| Rajasulochana et al. 2023 | National | Cross-Sectional | 1,351 (hospitalization) + 6,379 (outpatient) = 7,730 individuals | 8 | High |
| Rout & Choudhury 2018 | Odisha | Cross-Sectional | 284 inpatients (surveyed across two districts of Odisha | 7 | High |
| Kumar et al 2021 | National | Prospective | 1,275 patients (274 tier 2, 891 tier 3, 110 ileal perforation) | 6 | Moderate |
| Pati et al., 2014 | National | Cross-Sectional | 11,230 adults | 6 | Modreate |
| George et al., 2024 | National | Cross-Sectional/ Observational | 65,932 households (2014) and 113,823 households (2017–18).  153 medicines (2001–2017 approvals), with 32 analysed for brand-level price variation. | 7 | High |
| Bose & Dutta, 2018 | State Wise | Cross-Sectional | 65,932 households (333,104 individuals) in NSS 71st round + 73,868 households in NSS 60th round; analysis restricted to inpatient care episodes in TN, Rajasthan, and WB. | 8 | High |
| (Pati et al., 2021) | Odisha | Qualitative | 17 Urban Primary Health Centres (UPHCs | 7 | High |
| Singh et al., 2016) | Punjab/District | Cross-Sectional | 1,008 households (multistage cluster survey in Punjab) | 7 | High |
| Goswami & Anand, 2023, | National | Cross-Sectional | 65,562 individuals (8,429 with diabetes, 57,133 without diabetes) | 6 | Modrate |
| Nanda & Sharma, 2023, #) | National | Cross-Sectional | 1,216 (inpatient DM) + 6,527 (outpatient DM) = 7,743 diabetes patients (from 113,823 households / 555,115 individuals overall) | 7 | High |
| Singh et al., 2022, # | Rajsthan | Cross-Sectional | 3,270 respondents from urban poor households. | 8 | High |
| (Behera & Pradhan, 2021, #) | National | Cross-Sectional | 113,823 households (nationwide NSS 75th round) | 7 | low risk |
| Ambade. etal | National | Cross-Sectional | 43,781 inpatients + 8,914 outpatients = 52,695 individuals | 8 | High |
| Bose & Banerjee (2019) | India (National; NSS 71st round) | Analytical cross-sectional (secondary data) | 333,104 individuals | 8 | High quality |
| Tripathy & Prasad (2016) | India (National; NSS 71st round, 2014) | Analytical cross-sectional (secondary data) | 333,104 individuals (≈14,609 NCD hospitalisations) | 8 | High quality |
| Tripathy et al. (Year) | India (state / regional) | Cross-sectional study | Reported in study | 7 | High quality |
| Shahrawat& Rao (2012) | India (National; NSS 61st round) | Analytical cross-sectional (secondary data) | 124,644 households | 8 | High quality |
| Koul et al. (2019) | North India (Jammu & Kashmir) | Hospital-based analytical cross-sectional | 129 severe AECOPD patients | 7 | High quality |
| Jacob et al. (2023) | South India (North Chennai, Tamil Nadu) | Community-based analytical cross-sectional | 230 adults with diabetes | 7 | High quality |
| Prinja et al. (2012) | North India (Haryana, Punjab, Chandigarh) | Analytical cross-sectional (NSS 60th round) | ~3,305 households | 7 | High quality |
| Prinja et al. (2023) | India (6 states; cancer centres) | Multi-centre analytical cross-sectional | 12,148 cancer patients | 8 | High quality |
| Thakur et al. (2017) | East Delhi (resettlement colony) | Community-based longitudinal cost study | 150 adults with diabetes | 7 | High quality |
| Singh et al. (2016) | Punjab (SBS Nagar district) | Rapid analytical cross-sectional survey | 1,010 households (4,227 individuals) | 7 / 8 | High quality |

**S6: Meta-Analysis data**

**S6-A: Table - 5**: **OOP on medicine incurred by chronic patients**

| **Study** | **Published year** | **Study conducted Year** | **Chronic** | **Mean OOP Medicine CPI 2024** | **SD** | **n** | **Unit** |
| --- | --- | --- | --- | --- | --- | --- | --- |
| Swain et al. 2018 | 2018 | 2016 | Diabetes | 14622.9961 | 9953.769025 | 206 | INR per patient per year |
| Rawat et al. 2024 | 2024 | 2019 | Diabetes | 6789.201342 | 22905 | 843 | INR per patient per year |
| Thakur et al. 2017 | 2017 | 2015 | Diabetes | 3416.333601 | 4656.688051 | 150 | INR per patient per year |
| Ranjan et al. 2019 | 2019 | 2017 | Rheumatic disease | 13,387.84 | 25489.25466 | 3699 | INR per patient per year |
| Pati et al. 2021 | 2021 | 2019 | Hypertension | 1319.105947 | 967.607655 | 137 | INR per patient per year |
| Pati et al. 2021 | 2021 | 2019 | Cancer | 1607.676828 | 795.1496924 | 137 | INR per patient per year |
| Pati et al. 2021 | 2021 | 2019 | Diabetes | 1379.663705 | 983.5369788 | 137 | INR per patient per year |
| Pati et al. 2021 | 2021 | 2019 | APD | 1596.223513 | 840.5680109 | 137 | INR per patient per year |
| Pati et al. 2021 | 2021 | 2019 | Thyroid | 1566.866165 | 745.2553657 | 137 | INR per patient per year |
| Dehury et al. 2025 | 2025 | 2022 | Chronic Illness | 1853.200447 | 6331.43559 | 902 | INR per HOUSEHOLD |

**S6-B: Table-6: Chronic vs. Non-Chronic patients**

| **Study** | **Published year** | **Study conducted Year** | **Chronic** | **Mean OOP Medicine CPI 2024** | **SD** | **n** | **Unit** |
| --- | --- | --- | --- | --- | --- | --- | --- |
| Swain et al. 2018 | 2018 | 2016 | Diabetes | 14622.9961 | 9953.769025 | 206 | INR per patient per year |
| Rawat et al. 2024 | 2024 | 2019 | Diabetes | 6789.201342 | 22905 | 843 | INR per patient per year |
| Thakur et al. 2017 | 2017 | 2015 | Diabetes | 3416.333601 | 4656.688051 | 150 | INR per patient per year |
| Ranjan et al. 2019 | 2019 | 2017 | Rheumatic disease | 13,387.84 | 25489.25466 | 3699 | INR per patient per year |
| **Study** | **Published year** | **Study conducted Year** | **Non- Chronic** | **Mean OOP Medicine CPI 2024** | **SD** | **n** | **Unit** |
| Dror et al. 2008 | 2008 | 2005 | Cost of illness | 1015.099476 | 1,905.56 | 2232 | INR per patient |
| Rout & Choudhury 2018 | 2018 | 2017 | All inpatients | 2314.668 | 1763.151707 | 230 | INR per patient |
| Kumar et al. 2021 (Tier 1) | 2021 | 2019 | Enteric fever | 2867.278195 | 4760.366 | 274 | INR per patient per year |
| Kumar et al. 2021 (Tier 2) | 2021 | 2019 | Enteric fever | 12353.78634 | 19522.3599 | 891 | INR per patient per year |

**S6-C: Table- 7: OOPE on medicines as part of total OOP on over all healthcare**

| **Study** | **Year** | **Outcome** | **Events** | **Total** | **Proportion percent** |
| --- | --- | --- | --- | --- | --- |
| Basu et al. | 2020 | Any OOP on medicines | 194 | 375 | 51.7 |
| Swain et al. | 2018 | Fully paying OOP for medicines | 115 | 206 | 55.8 |
| Dror et al. | 2008 | Share of drugs in total OOP | 2114 | 4316 | 49 |

**S6-D: Table-8: Public vs private**

| **Study** | **Published year** | **Study conducted Year** | **Non- Chronic** | **Mean OOP Medicine CPI 2024** | **SD** | **n** | **Unit** | **Setting** |
| --- | --- | --- | --- | --- | --- | --- | --- | --- |
| Rajasulochana et al. 2023 | 2023 | 2017 | Hypertension | 2075.8 | 4678.04 | 653 | INR per inpatient episode | Public |
| Rajasulochana et al. 2023 | 2023 | 2017 | Hypertension | 7392.9866 | 11,056.67 | 667 | INR per inpatient episode | Private |
| Ranjan et al. 2019 | 2019 | 2017 | Rheumatic diseases | 4,545.36 | 13,335.75 | 780 | INR per inpatient episode | Public |
| Ranjan et al. 2019 | 2019 | 2017 | Rheumatic diseases | 882.42666 | 13,642.31 | 1174 | INR per inpatient episode | Private |

**S7: Sensitivity Analysis**
